# Atrial fibrillation, major bleeding, heart failure, and postoperative complications in patients undergoing isolated on-pump coronary artery bypass grafting in the northeast of Iran: A retrospective cohort study

**DOI:** 10.1097/MD.0000000000048646

**Published:** 2026-05-08

**Authors:** Mahin Nomali, Keyvan Salehi, Aryan Ayati, Amirhossein Tayebi, Keyvan Moghaddam, Soheil Mosallami Aghili, Soroosh Aminolsharieh Najafi, Fahimeh Valizadeh Shiran, Mahdis Nomali, Pedram Pirmoradian, Maryam Karimi Ghahfarokhi, Shiva Armani Moghadam, Mehdi Rayatnavaz, Mohammad Isaq Mohammadi, Gholamreza Roshandel

**Affiliations:** a Department of Biostatistics and Epidemiology, School of Health, Mazandaran University of Medical Sciences, Sari, Iran; b Cardiovascular Research Center, Mazandaran University of Medical Sciences, Sari, Iran; c School of Medicine, Shahid Beheshti University of Medical Sciences, Tehran, Iran; d University of California, San Francisco, CA; e Cardiovascular Research Center, Alborz University of Medical Sciences, Alborz, Iran; f Supervisory Department, Kordkuy Amiralmomenin Hospital, Golestan University of Medical Sciences, Gorgan, Iran; g Open Heart Intensive Care Unit, Kordkuy Amiralmomenin Hospital, Golestan University of Medical Sciences, Gorgan, Iran; h Cardioangiologisches Centrum Bethanien (CCB), Frankfurt Am Main, Medizinische Klinik III, Agaplesion Markus Krankenhaus, Frankfurt Am Main, Germany; i Department of Cardiology, Tehran Heart Center, Tehran University of Medical Sciences, Tehran, Iran; j Shafa Super-Specialized Cardiovascular Hospital, Gorgan, Iran; k School of Medicine, Isfahan University of Medical Sciences, Isfahan, Iran; l Department of Biostatistics, School of Allied Medical Sciences, Shahid Beheshti University of Medical Sciences, Tehran, Iran; m Heart Failure Research Center, Cardiovascular Research Institute, Isfahan University of Medical Sciences, Isfahan, Iran; n Department of Epidemiology and Biostatistics, School of Public Health, Tehran University of Medical Sciences, Tehran, Iran; o School of Nursing, Kabul University of Medical Sciences, Abu Ali Ibn Sina, Kabul, Afghanistan; p Golestan Research Center of Gastroenterology and Hepatology, Jorjani Clinical Sciences Research Institute, Golestan University of Medical Sciences, Gorgan, Iran.

**Keywords:** coronary artery bypass, Iran, postoperative complications, retrospective studies

## Abstract

Coronary artery bypass grafting (CABG) is one of the most performed cardiothoracic surgeries worldwide. The aim was to assess postoperative complications of patients undergoing CABG in the northeast of Iran. This was a 10-year, large-scale retrospective cohort study in Golestan Province. Kordkuy Heart Center of Amiralmomenin Hospital and Shafa Private Heart Center were the study settings, which were the only heart centers providing services to patients with cardiovascular diseases in Golestan Province. Adult patients of both genders who underwent isolated on-pump CABG procedures between 2007 and 2016 were included, and postoperative complications were extracted directly from patients’ hospital records. Out of 3720 surgeries performed, 3704 eligible patients were recruited, with a mean (standard deviation) age of 59.0 (9.8) years, of whom 73% were male. The postoperative complications included acute atrial fibrillation (AF; 8.2%), major bleeding (3.5%), heart failure (HF) (3.1%), pneumonia (2.0%), myocardial infarction (1.3%), acute kidney injury (1.0%), and stroke (0.3%). According to multivariable analyses, age (odds ratio [OR] 1.01, 95% confidence interval [CI] 1.001, 1.02; *P* .028), left ventricular ejection fraction (OR 0.97, 95% CI 0.96, 0.99; *P* .004), and preoperative β-blocker (OR 1.31, 95% CI 1.02, 1.68; *P* .034) were associated with new-onset AF. Cardiopulmonary bypass time (OR 1.003, 95% CI 1.001, 1.006; *P* .042) was the only associated factor with postoperative major bleeding. Chronic obstructive pulmonary disease (OR 6.73, 95% CI 3.98, 11.40; *P* < .001), preoperative β-blocker (OR 2.60, 95% CI 1.69, 3.97; *P* < .001), and preoperative angiotensin-converting-enzyme inhibitors (OR 1.56, 95% CI 1.04, 2.33; *P* .029) were the associated factors with postoperative HF. In conclusion, acute AF, major bleeding, HF, pneumonia, myocardial infarction, acute kidney injury, and stroke were postoperative complications observed in patients undergoing isolated on-pump CABG.

## 1. Introduction

Cardiovascular diseases (CVDs) are one of the major causes of mortality and disability worldwide, with an estimated 23 million deaths by 2030.^[1]^ In 2020, coronary artery disease made up a significant proportion of cardiovascular disease cases, accounting for 41.2% of CVD-related deaths in the United States.^[2]^ Despite advancements in the conventional medical treatment of coronary artery disease, revascularization remains necessary in many patients to restore adequate myocardial perfusion.

Although less invasive methods like percutaneous coronary intervention (PCI) are increasingly used,^[3,4]^ coronary artery bypass grafting (CABG) remains the standard of care for severe or complex cases.^[5]^ Despite improvements in surgical techniques and perioperative care, postoperative complications remain a major concern following isolated on-pump CABG.^[6,7]^ Postoperative complications have been reported at about 41 percent,^[8]^ which contribute to increased morbidity, mortality, extended hospital stay, and higher healthcare costs.^[9–11]^ On the other hand, according to the Golestan Cohort Study, a large prospective study conducted in northeast Iran, ischemic heart disease accounts for 33.9% of premature deaths in the region, and 30.5% of deaths occur in individuals under 60 years of age,^[12]^ with a recent analysis from the same cohort additionally highlighting significant ethnic disparities in major adverse cardiac and cerebrovascular events and postoperative outcomes following CABG.^[13]^

Due to a lack of large-scale, region-specific data from northeastern Iran, a region with distinct demographic and clinical features that may affect complications, we evaluated the postoperative complications and associated factors in patients undergoing isolated on-pump CABG through a 10-year study in the northeast of Iran, which provides valuable insights to improve patient management and optimize surgical outcomes.

## 2. Materials and methods

### 2.1. Study design

This 10-year large-scale retrospective cohort study was conducted in Golestan Province. The protocol was approved by the Institutional Review Board of Golestan University of Medical Sciences on July 26, 2016 (approval ID. 950505.06) and the research ethics committee on September 4, 2016 (approval ID. IR.GOUMS.REC.1395.137).^[13–15]^ Due to the retrospective nature of the data collection method and the use of patients’ health records, informed consent was not obtained.

### 2.2. Setting

Our study was conducted in 2 heart centers in the northeastern region of Iran; these were the only centers serving patients with CVDs in Golestan Province during the study period. The first center was a tertiary hospital with 141 beds, located in the western part of Golestan Province, affiliated with a Medical Sciences University. Established in 2003, it served as the sole heart center until 2015, offering services such as coronary angiography, angioplasty, and percutaneous interventions, and open-heart surgeries to patients not only from Golestan Province but also from other regions and international patients from Iraq, Tajikistan, the Republic of Azerbaijan, Turkmenistan, and Kazakhstan. The second heart center, a private hospital in the region, initiated performing CABG surgeries in 2015 with a surgical team and operative CABG technique similar to the first center.

### 2.3. Participants

The study included adult male and female patients who underwent isolated CABG procedures between 2007 and 2016. Data were extracted from the clinical records of these patients. Individuals under 18 years of age and those who underwent cardiac surgeries other than isolated on-pump CABG were excluded.

All patients were treated using a standardized surgical technique. After anesthesia induction, a median sternotomy and aorta-right atrial cannulation were performed to establish cardiopulmonary bypass (CPB). Revascularization was carried out during aortic cross-clamping and cardioplegic arrest for all patients. This was achieved by occluding the ascending aorta and perfusing the heart with a cardioplegia solution. St. Thomas crystalloid cardioplegia solution, consisting of sodium, potassium, magnesium, calcium, bicarbonate, and procaine added to Ringer’s solution, was used.^[16]^ The solution was infused through a cardioplegic catheter inserted into the aorta proximal to the cross-clamp to initiate immediate chemical arrest upon aortic cross-clamping. The cold hyperkalemic crystalloid solution was then infused antegradely, with a volume not exceeding 1000 mL. If there was evidence of resumption of electrical heart activity or prolonged ischemic time, 1 or more infusions of 300 to 500 mL of the cardioplegic solution might be administered. In cases of myocardial revascularization, the aortic cross-clamp was removed after completing the distal anastomoses, and the heart was reperfused while conducting the proximal anastomoses using a partial occlusion clamp. Alternatively, the proximal grafts were performed after the distal grafts were completed, with the cross-clamp still in place (the single-clamp technique).^[16]^

### 2.4. Variables and measurement

The study variables encompassed demographic and clinical characteristics, paraclinical evaluations, and operative and postoperative characteristics. Data were collected by completing a study checklist based on patients’ clinical records. Demographic characteristics included age, sex, ethnicity, education, marital status, body mass index, alcohol drinking (self-reported), smoking (self-reported), and opium consumption (self-reported). Body mass index categories followed the Centers for Disease Control and Prevention guidelines, classifying individuals as underweight (below 18.5), average (18.5–24.9), overweight (25–29.9), and obese (30 and above).^[17]^ Clinical factors comprised systolic blood pressure (mm Hg), heart rate (beats per minute) at admission, family history of CVDs, past medical history of myocardial infarction (MI), PCI, CABG, valve surgery, cardiac arrest, cardiopulmonary resuscitation, stroke, unstable angina, comorbidities such as diabetes mellitus, hypertension (HTN), hyperlipidemia, chronic kidney disease, chronic obstructive pulmonary disease (COPD), gastrointestinal disorders, malignancy, peripheral vascular disorders and psychological disorders, and medications including β-blockers, angiotensin-converting enzyme (ACE) inhibitors, calcium channel blockers, diuretics, statins, aspirin, vasodilators, and warfarin. Paraclinical evaluations included blood tests, coronary artery angiography, and transthoracic echocardiography performed before the operation, with results documented in the checklist. Information on operative characteristics was obtained from the surgeon’s and perfusionist’s reports, the open-heart ICU nursery sheet review, and the surgeon’s written or call orders. Postoperative complications, such as bleeding, acute onset of atrial fibrillation (AF), heart failure (HF), pneumonia, MI, acute kidney injury (AKI), and stroke, were defined as per the criteria outlined in Table S1, Supplemental Digital Content. It is essential to note that this study relied on clinical record-based data collection, with information extracted directly from patients’ hospital records.

### 2.5. Statistical analysis

In this study, we utilized the available data approach to analyze all observed data. Categorical variables were presented as numbers and percentages, while continuous variables with a normal distribution were reported as means and standard deviations. The median and interquartile range (IQR) were used for variables without a normal distribution. A logistic regression analysis was conducted to identify factors associated with postoperative complications. Both univariate and multivariable logistic regression models were employed. Variables were initially screened in univariate analysis, and those with a *P* value < .2, along with clinically relevant variables based on expert knowledge, were included in the multivariable models. Factors with a *P* value below .05 in the multivariable analysis were considered statistically significant and reported as odds ratios (ORs) with 95% confidence intervals (CIs). To run logistic regressions identifying factors associated with each outcome, we adhered to the ten events per variable rule of thumb.

The statistical software package STATA/IC version 14.2 (Stata Corp LP, College Station) was used for the statistical analyses.

## 3. Results

### 3.1. Participants

Between 2007 and 2016, 3720 patients underwent isolated on-pump CABG surgery in the study centers. After excluding 16 patients under 18 years, 3704 eligible patients were included in the study. Of these, 3341 patients (90%) were from the Kordkuy Heart Center of Amiralmomenin Hospital, while 363 patients (10%) were from the Shafa Private Heart Center.

### 3.2. Descriptive data

Table 1 summarizes the baseline characteristics of patients who underwent CABG. The mean age of the 3704 patients was 59.0 years, with 14.2% of patients being 70 years or older. Most patients were male (63%), married (87.6%), overweight (43.9%), of non-Turkman ethnicity (92%), and with an elementary school education (43%). Regarding habits, 29.4% had a history of opium consumption, 14% were smokers, and 1.1% reported alcohol drinking. Clinically, patients had a mean SBP of 130.69 mm Hg and a mean heart rate of 77.4 bpm. A positive family history of CVDs was prevalent in 71.7% of the cohort. Common comorbidities included HTN (58.5%), hyperlipidemia (57.3%), and diabetes (48%). Prior medical history revealed that 11% had unstable angina, 6% had a history of MI, and a small number had undergone previous procedures, such as PCI (1%). Regarding medication use before surgery, 84.4% of patients were on aspirin, 77.5% on statins, and 50% on vasodilators. Additionally, 48.2% were taking β-blockers, 42.1% were on ACE inhibitors, and 12% were using calcium channel blockers.

**Table 1:** Baseline characteristics of studied patients underwent isolated on-pump coronary artery bypass grafting.

Characteristics	Overall value	
Demographic characteristics		
Age (yr), mean (SD) (n = 3704)	59.0 (9.8)	
≥70 yr, n/N (%)		525/3704 (14.2)
Female gender, n/N (%)	1368/3704 (37.0)	
Marital status, n/N (%)		
Single, n/N (%)	122/3018 (4.0)	
Married, n/N (%)	2643/3018 (87.6)	
Widowed, n/N (%)	241/3018 (8.0)	
Divorced, n/N (%)	12/3018 (0.4)	
Education		
Illiterate, n/N (%)	758/2948 (27.0)	
Elementary, n/N (%)	1267/2948 (43.0)	
High school, n/N (%)	701/2948 (24.0)	
Academic, n/N (%)	195/2948 (6.0)	
Ethnicity		
Turkman, n/N (%)	301/3632 (8.0)	
Non-Turkman, n/N (%)	3331/3632 (92.0)	
BMI (kg/m^2^), mean (SD) (n = 3670)	27.65 (4.66)	
Underweight, n/N (%)	54/3670 (1.4)	
Normal, n/N (%)	983/3670 (26.8)	
Overweight, n/N (%)	1610/3670 (43.9)	
Obese, n/N (%)	1023/3670 (27.9)	
Habits		
Opium consumption, n/N (%)	985/3355 (29.4)	
Cigarette smoking, n/N (%)	464/3324 (14.0)	
Alcohol drinking, n/N (%)	36/3316 (1.1)	
Clinical characteristics		
SBP (mm Hg), mean (SD) (n = 3637)	130.69 (19.98)	
HR (bpm), mean (SD) (n = 3632)	77.39 (11.32)	
Family history of CVDs, n/N (%)	2598/3625 (71.7)	
Past medical history		
MI, n/N (%)	206/3704 (6.0)	
PCI, n/N (%)	24/3700 (1.0)	
CABG, n/N (%)	10/3504 (0.3)	
Valve surgery, n/N (%)	2/3504 (0.1)	
Cardiac arrest and CPR, n/N (%)	4/3503 (0.1)	
Stroke, n/N (%)	2/3503 (0.1)	
Unstable angina, n/N (%)	385/3504 (11.0)	
Comorbidities		
DM, n/N (%)	1730/3605 (48.0)	
HTN, n/N (%)	2094/3579 (58.5)	
Hyperlipidemia, n/N (%)	2083/3637 (57.3)	
CKD, n/N (%)	228/3522 (6.5)	
COPD, n/N (%)	208/3532 (6.0)	
GI disorders, n/N (%)	240/3504 (7.0)	
Malignancy, n/N (%)	4/3504 (0.2)	
Peripheral vascular disorders	3/3504 (0.1)	
Psychological disorders	10/3503 (0.3)	
Medications		
Beta-blocker, n/N (%)	1708/3540 (48.2)	
ACE inhibitor, n/N (%)	1478/3507 (42.1)	
Calcium channel blocker (CCB), n/N (%)	414/3497 (12.0)	
Diuretic, n/N (%)	177/3508 (5.0)	
Statin, n/N (%)	2860/3688 (77.5)	
Aspirin, n/N (%)	3113/3688 (84.4)	
Vasodilator, n/N (%)	1803/3623 (50.0)	
Warfarin, n/N (%)	16/3494 (0.5)	

BMI = body mass index, bpm = beats per minute, CABG = coronary artery bypass graft, CKD = chronic kidney disease, COPD = chronic obstructive pulmonary disease, CPR = cardiopulmonary resuscitation, CVD = cardiovascular disease, DM = diabetes mellitus, GI = gastrointestinal, HR = heart rate, HTN = hypertension, MI = myocardial infarction, PCI = percutaneous coronary intervention, SBP = systolic blood pressure, SD = standard deviation.

Table S2, Supplemental Digital Content, shows the results of both invasive and noninvasive paraclinical evaluations of study patients. The median (IQR) values for total cholesterol and fasting plasma glucose were 210 mg/dL (169–270) and 110 mg/dL (93.0–141.0), respectively, while the mean (standard deviation) for low-density lipoprotein was 150 (44.4) mg/dL. The angiographic evaluation indicated that the majority of patients (72%) had 3-vessel disease. Also, the left anterior descending artery (95.4%), right coronary artery (79.4%), and left circumflex artery (76.2%) were the most common infarct-related arteries. According to the transthoracic echocardiography, the median (IQR) of left ventricular ejection fraction (LVEF) was 50% (45, 50%).

Operative and postoperative measures of the study patients are summarized in Table S3, Supplemental Digital Content. Only 4.6% of patients underwent an emergent operation, and 6.5% of them underwent CABG surgery after MI. The medians (IQRs) of bypass time and clamp time were 110 (58–130) minutes and 39 (30–50) minutes, respectively. The median (IQR) of the number of grafts was 3 (3–4), and the saphenous artery (93%) and left internal mammary artery (90.3%) grafts were the most commonly used grafts. The median (IQR) length of stay in the open-heart ICU was 4 days (3–5 days). Intravenous trinitroglycerin (77%) and inotrope (30.5%) were the most applied postoperative measures.

### 3.3. Outcome data

Table S4, Supplemental Digital Content, summarizes the postoperative complications of patients undergoing isolated on-pump CABG. The most common postoperative complications were acute AF (8.2%), major bleeding (3.5%), HF (3.1%), pneumonia (2.0%), MI (1.3%), AKI (1.0%), and stroke (0.3%). Patients aged ≥70 years had a higher proportion of acute AF (12.2% vs 7.5%, *P* < .001), pneumonia (3.6% vs 1.6%, *P* = .002), and stroke (0.9% vs 0.2%, *P* .006) compared with those <70 years. Gender analysis showed males had a higher proportion of bleeding (4.0% vs 3.0%, *P* .048) and pneumonia (2.2% vs 1.3%, *P* .05) than females. Other complications, such as stroke and MI, did not differ significantly by gender.

### 3.4. Main results

In this study, univariate and multivariable analyses of factors associated with the most common postoperative complications, including acute onset of AF (Table 2), major bleeding (Table 3), and HF (Table 4), have been evaluated. Table 2 indicates that age (OR 1.01, 95% CI 1.001, 1.02; *P* .028), LVEF (OR 0.97, 95% CI 0.96, 0.99; *P* .004), and preoperative β-blocker (OR 1.31, 95% CI 1.02, 1.68; *P* .034) were the associated factors with new onset of AF after CABG. Table 3 shows that CPB time (OR 1.003, 95% CI 1.001, 1.006; *P* .042) was the only associated factor with postoperative major bleeding. According to Table 4, COPD (OR 6.73, 95% CI 3.98, 11.40; *P* < .001), preoperative β-blocker (OR 2.60, 95% CI 1.69, 3.97; *P* < .001), and preoperative ACE inhibitor (OR 1.56, 95% CI 1.04, 2.33; *P* .029) were the associated factors with HF after isolated on-pump CABG surgery.

**Table 2:** Univariate and multivariable analyses of factors associated with atrial fibrillation after isolated on-pump coronary artery bypass grafting.

Variables	Unadjusted analysis		Adjusted analysis	
	OR (95% CI)	*P* value	OR (95% CI)	*P* value
Age, yr	1.02 (1.01, 1.03)	.001*	1.01 (1.001, 1.02)	.028†
Male gender	0.96 (0.75, 1.23)	.778	–	–
LVEF, %	0.97 (0.95, 0.98)	<.001*	0.97 (0.96, 0.99)	.004†
CPB time, min	0.99 (0.99, 1.00)	.842	–-	–
Clamp time, min	0.99 (0.99, 1.00)	.824	–	–
RCA stenosis	0.93 (0.69, 1.24)	.626	–	–
Grafts > 3	1.09 (0.86, 1.40)	.447	–	–
Preoperative β-blocker	1.37 (1.07, 1.76)	.011*	1.31 (1.02, 1.68)	.034†
Preoperative urea	1.01 (1.002, 1.02)	.006*	1.01 (1.00, 1.01)	.050
Preoperative creatinine	0.98 (0.90, 1.06)	.702	–	–

CI = confidence interval, CPB = cardiopulmonary bypass time, LVEF = left ventricular ejection fraction, min = minute, OR = odds ratio, RCA = right coronary artery.

* Probability value < .2, variables included in the multivariable analysis.

† Probability value < .05, considered as statistically significant.

**Table 3:** Univariate and multivariable analyses of factors associated with major bleeding after isolated on-pump coronary artery bypass grafting.

Variables	Unadjusted analysis		Adjusted analysis	
	OR (95% CI)	*P* value	OR (95% CI)	*P* value
Age, yr	1.00 (0.98, 1.02)	.812	–	–
Male gender	1.47 (1.001, 2.17)	.049*	1.47 (0.94, 2.30)	.094
BMI, kg/m^2^	1.00 (0.96, 1.04)	.967	–	–
Diabetes	0.93 (0.65, 1.32)	.686	–	–
HTN	1.37 (0.94, 1.98)	.103*	1.25 (0.81, 1.94)	.307
Preoperative hemoglobin	0.99 (0.98, 1.01)	.104*	1.00 (0.98, 1.02)	.448
Preoperative platelet count	1.00 (0.99, 1.02)	.321	–	–
Emergent operation	0.66 (0.24, 1.81)	.421	–	–
CPB time, min	1.003 (1.002, 1.01)	.036*	1.003 (1.001, 1.006)	.042†
Clamp time, min	1.00 (0.99, 1.00)	.973	–	–
Preoperative aspirin	0.89 (0.55, 1.41)	.613	–	–
LMCA stenosis	1.02 (0.58, 1.80)	.935	–	–

CI = confidence interval, BMI = body mass index, CPB = cardiopulmonary bypass time, HTN = hypertension, LMCA = left main coronary artery, OR = odds ratio.

* Probability value < .2, variables included in the multivariable analysis.

† Probability value < .05, considered as statistically significant.

**Table 4:** Univariate and multivariable analyses of factors associated with heart failure after isolated on-pump coronary artery bypass grafting.

Variables	Unadjusted analysis		Adjusted analysis	
	OR (95% CI)	*P* value	OR (95% CI)	*P* value
Age, yr	1.01 (0.99, 1.03)	.184*	0.99 (0.97, 1.01)	.518
Female gender	1.07 (0.73, 1.57)	.709	–	–
Opium consumption	1.46 (0.99, 2.16)	.056*	1.29 (0.86, 1.94)	.211
Smoking	0.73 (0.39, 1.34)	.315	–	–
Diabetes	0.75 (0.51, 1.09)	.138*	0.73 (0.49, 1.08)	.121
HTN	0.99 (0.68, 1.45)	.982	–	–
CKD	1.6 (0.85, 3.03)	.147*	1.20 (0.60, 2.30)	.651
COPD	5.31 (3.35, 8.44)	<.001*	6.73 (3.98, 11.40)	<.001†
Preoperative β-blocker	2.08 (1.40, 3.08)	<.001*	2.60 (1.69, 3.97)	<.001†
Preoperative ACE inhibitor	2.01 (1.37, 2.95)	<.001*	1.56 (1.04, 2.33)	.029†

β-blocker = beta-blocker, CI = confidence interval, ACE = angiotensin-converting enzyme, CKD = chronic kidney disease, COPD = chronic obstructive pulmonary disease, HTN = hypertension, OR = odds ratio.

* Probability value < .2, variables included in the multivariable analysis.

† Probability value < .05, considered as statistically significant.

## 4. Discussion

This was a 10-year, large-scale study of 3704 patients who underwent CABG surgery in northeast Iran, comprehensively examining demographic and clinical characteristics, paraclinical evaluations, operative characteristics, and postoperative complications. Additionally, the study investigated the associated factors for the most common complications, aiming to provide comprehensive insights specific to the northeast of the Iranian population.

### 4.1. Demographic and clinical characteristics

The mean age of the study patients was 59 years, which was consistent with previous Iranian studies^[18,19]^ but younger than cohorts from other American and European populations.^[20,21]^ A high prevalence of overweight and obesity was observed, exceeding rates reported in comparable studies.^[22]^ Regarding comorbidities, diabetes mellitus and HTN were highly prevalent, with diabetes notably higher than reported in many Asian and Western studies.^[18,23–25]^ Although diabetes is a well-established risk factor for certain postoperative complications, such as HF, current evidence does not support its role as a risk factor for others, such as major bleeding.^[15,26]^ Hyperlipidemia had a high frequency among the patients, which is considerably higher than similar studies in Iran and Western countries.^[23,27]^ The demographic and paraclinical profile of our cohort, marked by high proportions of obesity, diabetes, and hyperlipidemia, reflects a population at elevated risk for postoperative complications.

### 4.2. Operative characteristics and postoperative complications

The median CPB time was 110 minutes, aligning with the reported value in international studies.^[28]^ Similarly, the median number of grafts was 3, and the left internal mammary artery and saphenous veins were predominantly used, consistent with standard CABG practice worldwide.^[29–31]^ This study focused on in-hospital postoperative complications in patients undergoing isolated on-pump CABG. Mortality outcomes were excluded, as they were previously reported.^[14]^ Among the 3704 patients analyzed, AF, major bleeding, and HF were the most common complications, followed by pneumonia, MI, AKI, and stroke.

AF was the most common complication. In our multivariable analysis, advanced age, reduced LVEF, and preoperative β-blocker use were independently associated with new-onset AF. In addition, our findings revealed a 5% increase in AF risk per 5-year age increment. In a similar study, Zaman et al indicated that signal-averaged P-wave duration >155 ms, advanced age, and male sex were independent predictors of AF. In their study, the odds of new-onset AF increased by 53% for every 5-year increase in age.^[32]^ This discrepancy may reflect differences in population characteristics, comorbidities, or perioperative care. Interestingly, our study found that preoperative β-blocker use increased the odds of AF by 31%, higher than the 9% reported by Brinkman et al’s study.^[33]^ About postoperative major bleeding, CPB time was the only significant predictor among the identified risk factors. Previous studies have also implicated advanced age and low postoperative platelet counts in bleeding risk.^[34]^ However, these were not significant in our analysis. The direct link between CPB duration and bleeding risk underscores the importance of minimizing pump time when possible.

We found that COPD, preoperative β-blocker use, and preoperative ACE inhibitor use were significantly associated with an increased risk of postoperative HF. Notably, preoperative β-blocker use was associated with more than a twofold increase in HF risk. It is important to note that all patients with a prior history of HF were excluded, allowing us to assess factors associated with new-onset postoperative HF. As highlighted in a systematic review by Thaper and Kulik,^[35]^ the benefit of preoperative β-blocker use in CABG patients without prior MI or congestive HF remains controversial, with several large observational studies failing to show improvements in perioperative morbidity or mortality. Our results suggest that preoperative β-blocker and ACE inhibitor use in CABG candidates may be associated with increased vulnerability to postoperative HF, possibly due to adverse hemodynamic effects or impaired compensatory mechanisms during surgery. This highlights the need for further prospective studies to evaluate the impact of these medications in CABG patients, especially concerning perioperative dosing, drug type, and patient-specific characteristics.

Besides the strengths of this study, it had a major limitation in its nature. In the present study, hospital records were the only source of data collection, and data related to some variables for some patients may not have been recorded, which can be considered a major study limitation. In order to make the most use of the available data, we reported the number of patients for each variable and its categories separately.

In conclusion, this study identified several postoperative complications, including acute AF, major bleeding, HF, pneumonia, MI, AKI, and stroke in patients undergoing isolated on-pump CABG surgery. Notably, advanced age, reduced LVEF, and preoperative β-blocker use were significantly associated with new-onset AF, while prolonged CPB time was linked to major bleeding. Additionally, COPD and the use of preoperative β-blockers and ACE inhibitors were associated factors with postoperative HF. These findings highlight the importance of careful preoperative assessment and tailored perioperative management to mitigate the risk of adverse outcomes and improve patient prognosis following CABG surgery.

## Acknowledgments

The authors would like to thank the Research and Technology Deputy of Golestan University of Medical Sciences for their support in appraising the study protocol, facilitating the study’s conduct, and financial support. At the end, we would like to thank the Kordkuy Heart Center of Amiralmomenin Hospital and Shafa Super-Specialized Cardiovascular Hospital for making this study possible through their valuable cooperation during the study period.

## Author contributions

**Conceptualization:** Mahin Nomali, Keyvan Salehi, Aryan Ayati, Amirhossein Tayebi.

**Funding acquisition:** Gholamreza Roshandel.

**Data curation:** Mahin Nomali.

**Formal analysis:** Mahin Nomali, Maryam Karimi Ghahfarokhi.

**Investigation:** Mahin Nomali, Keyvan Moghaddam, Soheil Mosallami Aghili, Mahdis Nomali.

**Methodology:** Mahin Nomali, Gholamreza Roshandel.

**Project administration:** Mahin Nomali, Mahdis Nomali, Keyvan Moghaddam, Soheil Mosallami Aghili, Gholamreza Roshandel.

**Resources:** Gholamreza Roshandel.

**Supervision:** Gholamreza Roshandel.

**Validation:** Mahin Nomali, Soroosh Aminolsharieh Najafi, Fahimeh Valizadeh Shiran, Gholamreza Roshandel, Mehdi Rayatnavaz.

**Visualization:** Mahin Nomali, Maryam Karimi Ghahfarokhi.

**Writing – original draft:** Mahin Nomali, Keyvan Salehi, Aryan Ayati, Amirhossein Tayebi, Shiva Armani Moghadam.

**Writing – review & editing:** Mahin Nomali, Keyvan Salehi, Amirhossein Tayebi, Soroosh Aminolsharieh Najafi, Fahimeh Valizadeh Shiran, Gholamreza Roshandel, Mohammad Isaq Mohammadi, Mehdi Rayatnavaz.








