# Supplementary material for: Atrial fibrillation, major bleeding, heart failure, and postoperative complications in patients undergoing isolated on-pump coronary artery bypass grafting in the northeast of Iran: A retrospective cohort study
Source: Medicine (Baltimore). 2026 May 8;105(19):e48646. doi: 10.1097/MD.0000000000048646 (PMC13166559; doi:10.1097/MD.0000000000048646)
Supplement: Supplementary file 1 [file medi-105-e48646-s001.docx]

**Supplementary**

**Table S1.** Definition of postoperative complications

| **Postoperative complications** | **Definition** |
| --- | --- |
| **Myocardial infarction (MI)** | Clinical evidence of acute myocardial ischemia and detection of a rise in cardiac troponin levels with at least one value above the 99th percentile upper limit and at least one of the following:   - - Symptoms of myocardial ischemia   - New ischemic electrocardiogram changes   - Development of pathological Q waves (1) |
| **Stroke** | It was defined as a new focal neurological deficit that lasted more than 24 hours and was confirmed by imaging (2) |
| **Major bleeding** | Postoperative bleeding was defined as bleeding that necessitated reoperation and was documented in the patient’s medical record. |
| **Postoperative AF** | It was defined as a new onset of AF, which was detected by Electrocardiograms read by the attending cardiologists |
| **Heart failure** | It was defined as significant decrease in ejection fraction compared to baseline, which was diagnosed by the attending cardiologists |
| **Pneumonia** | Diagnosed by pulmonologist, which was documented in the patient’s medical record. |
| **Acute kidney injury** | It was defined as an increase in serum creatinine concentration of 0.3 mg/dL or greater (3, 4) |

**References**:

1. Thygesen K, Alpert JS, Jaffe AS, Chaitman BR, Bax JJ, Morrow DA, et al. Fourth Universal Definition of Myocardial Infarction (2018). Circulation. 2018 Nov 13;138(20):e618–51.
2. Chandrasekhar J, Marley P, Allada C, McGill D, O’Connor S, Rahman M, et al. Symptom-to-Balloon Time is a Strong Predictor of Adverse Events Following Primary Percutaneous Coronary Intervention: Results From the Australian Capital Territory PCI Registry. Heart Lung Circ. 2017 Jan;26(1):41–8.
3. Benedetto U, Angeloni E, Luciani R, Refice S, Stefanelli M, Comito C, et al. Acute kidney injury after coronary artery bypass grafting: does rhabdomyolysis play a role. J Thorac Cardiovasc Surg. 2010;140(2):464–70.
4. Chang TI, Leong TK, Boothroyd DB, Hlatky MA, Go AS. Acute kidney injury after CABG versus PCI: an observational study using 2 cohorts. J Am Coll Cardiol. 2014;64(10):985–94
