# Supplementary material for: Atrial fibrillation, major bleeding, heart failure, and postoperative complications in patients undergoing isolated on-pump coronary artery bypass grafting in the northeast of Iran: A retrospective cohort study
Source: Medicine (Baltimore). 2026 May 8;105(19):e48646. doi: 10.1097/MD.0000000000048646 (PMC13166559; doi:10.1097/MD.0000000000048646)
Supplement: Supplementary file 2 [file medi-105-e48646-s002.docx]

**Supplementary**

**Table S3.** Operative and postoperative measures of studied patients undergoing isolated on-pump coronary artery bypass grafting

| **Operative characteristics** | | | | **Overall Value** |
| --- | --- | --- | --- | --- |
|  | Emergent operation, n/N (%) | | | 169/3704 (4.6) |
|  | Post MI surgery, n/N (%) | | | 160/2481 (6.5) |
|  | CPB time (min) [Median (IQR)] (n=2250) | | | 110 (58-130) |
|  | Clamp time (min) ) [Median (IQR)] (n=2250) (n= 2248) | | | 39 (30-50) |
|  | Number of grafts [Median (IQR)] (n= 3329) | | | 3 (3-4) |
|  | 1 or 2 | | | 550/ 3329 (16.5) |
|  | 3 | | | 1198/3329 (36.0) |
|  | 4 | | | 1461/3329 (44.0) |
|  | ≥5 | | | 120/3329 (3.5) |
|  | Graft type | | |  |
|  |  | | LIMA graft, n/N (%) | 3294/3647 (90.3) |
|  |  | | Saphenous graft, n/N (%) | 3390/3648 (93.0) |
|  |  | | Radial graft, n/N (%) | 13/ 3461 (0.4) |
|  | Number of anastomosis | | |  |
|  |  | | Distal anastomosis, [Median (IQR)] (n= 3191) | 3 (3-4) |
|  |  | | Proximal anastomosis, [Median (IQR)] (n=3086) | 2 (2-3) |
| **Postoperative measures** | | | |  |
|  |  | Open heart ICU length of stay (day) [Median (IQR)] (n= 3414) | | 4 (3-5) |
|  |  | IABP, n/N (%) | | 74/3424 (2.2) |
|  |  | Pacemaker, n/N (%) | | 35/ 3424 (1.0) |
|  |  | IV inotrope, n/N (%) | | 1077/3526 (30.5) |
|  |  | IV TNG, n/N (%) | | 2818/ 3673 (77.0) |

IQR: interquartile range; MI: Myocardial infarction; CBP: Cardiopulmonary bypass; LIMA: left internal mammary artery; ICU: Intensive care unit; IABP: Intra aortic balloon pump; IV: intravenous; TNG: Trinitroglycerin
