# Supplementary material for: Atrial fibrillation, major bleeding, heart failure, and postoperative complications in patients undergoing isolated on-pump coronary artery bypass grafting in the northeast of Iran: A retrospective cohort study
Source: Medicine (Baltimore). 2026 May 8;105(19):e48646. doi: 10.1097/MD.0000000000048646 (PMC13166559; doi:10.1097/MD.0000000000048646)
Supplement: Supplementary file 3 [file medi-105-e48646-s003.docx]

**Supplementary**

**Table S4.** Postoperative complications of patients undergoing isolated on-pump coronary artery bypass grafting

|  | **Postoperative complications, n (%)** | | Overall  (n=3704) | Age <70 years  (n=3179) | Age ≥70 years  (n=525) | P value | Male  (n=2336) | Female  (n=1368) | P value |
| --- | --- | --- | --- | --- | --- | --- | --- | --- | --- |
|  |  | Acute atrial fibrillation | 304 (8.2) | 240 (7.5) | 64 (12.2) | <0.001* | 194 (8.3) | 110 (8.0) | 0.778 |
|  |  | Major Bleeding | 129 (3.5) | 107 (3.0) | 22 (4.0) | 0.340 | 92 (4.0) | 37 (3.0) | 0.048* |
|  |  | Heart failure | 114 (3.1) | 92 (3.0) | 22 (4.1) | 0.111 | 70 (3.0) | 44 (3.2) | 0.709 |
|  |  | Pneumonia | 70 (2.0) | 51 (1.6) | 19 (3.6) | 0.002* | 52 (2.2) | 18 (1.3) | 0.05* |
|  |  | Myocardial infarction | 47 (1.3) | 39 (1.2) | 8 (1.5) | 0.573 | 32 (1.4) | 15 (1.1) | 0.473 |
|  |  | Acute kidney injury | 25 (1.0) | 20 (0.6) | 5 (0.9) | 0.402 | 16 (0.7) | 9 (0.6) | 0.923 |
|  |  | Stroke | 12 (0.3) | 7 (0.2) | 5 (0.9) | 0.006* | 6 (0.3) | 6 (0.4) | 0.348 |

*Probability value <0.05, considered as statistically significant
