# Supplementary material for: Atrial fibrillation, major bleeding, heart failure, and postoperative complications in patients undergoing isolated on-pump coronary artery bypass grafting in the northeast of Iran: A retrospective cohort study
Source: Medicine (Baltimore). 2026 May 8;105(19):e48646. doi: 10.1097/MD.0000000000048646 (PMC13166559; doi:10.1097/MD.0000000000048646)
Supplement: Supplementary file 4 [file medi-105-e48646-s004.docx]

**Supplementary**

**Table S2.** Paraclinical (invasive and noninvasive) evaluations of studied patients undergoing isolated on-pump coronary artery bypass grafting

| **Paraclinical evaluations** | | | | | **Overall Value** |
| --- | --- | --- | --- | --- | --- |
| **Blood test** | | | | |  |
|  | **Lipid profile** | | | |  |
|  |  | | | Total cholesterol (mg/dl) [Median (IQR)] (n=3376) | 210.0 (169.0-270.0) |
|  |  | | | HDL (mg/dl) [Median (IQR)] (n=3543) | 40.0 (34.0 – 46.0) |
|  |  | | | LDL (mg/dl) [Mean (SD)] (n=3537) | 150.0 (44.4) |
|  | **CBC diff.** | | | |  |
|  |  | WBC [Median (IQR)] (n=3653) | | | 7300 (6100-8800) |
|  |  | RBC [Median (IQR)] (n=3631) | | | 4.4 (4.0-5.2) |
|  |  | Platelets (count) [Median (IQR)] (n=3693) | | | 235.0 (187.0-274.0) |
|  |  | Hemoglobin (g/dl) [Median (IQR)] (n=3695) | | | 12.6 (11.6-13.8) |
|  |  | Hematocrit (%)[Median (IQR)] (n=3696) | | | 38.5 (35.5-41.5) |
| **Other tests** | | | | |  |
|  |  | ALT (U/L) [Median (IQR)] (n=3600) | | | 29.0 (20.0-36.0) |
|  |  | AST (U/L) [Median (IQR)] (n=3603) | | | 28.0 (18.0-36.0) |
|  |  | ALP (U/L) [Median (IQR)] (n=3547) | | | 195 (156-222) |
|  |  | Albumin (g/dl) [Median (IQR)] (n=676) | | | 5.0 (4.1-5.1) |
|  |  | Total Bilirubin (mg/dl) [Median (IQR)] (n=3554) | | | 1.0 (0.8-1.1) |
|  |  | Fasting plasma glucose[Median (IQR)] (mg/dl) (n=3670) | | | 110.0 (93.0-141.0) |
|  |  | BUN (mg/L) [Median (IQR)] (n=3648) | | | 35.0 (29.0-43.0) |
|  |  | Creatinine (mg/L) [Median (IQR)] (n=3676) | | | 1.02 (0.9-1.2) |
|  |  | CRP (mg/L) [Median (IQR)] (n=188) | | | 1.0 (1.0-2.0) |
| **Angiographic characteristics** | | | | |  |
|  | Number of diseased vessels | | | |  |
|  |  | | One, n/N (%) | | 227/3501 (6.0) |
|  |  | | Two, n/N (%) | | 761/3501 (22.0) |
|  |  | | Three, n/N (%) | | 2513/3501 (72.0) |
|  | Infarct-related artery | | | |  |
|  |  | LMCA, n/N (%) | | | 390 /3496 (12.0) |
|  |  | LCX, n/N (%) | | | 2786 /3656 (76.2) |
|  |  | LAD, n/N (%) | | | 3505/3675 (95.4) |
|  |  | RCA, n/N (%) | | | 2884/3632 (79.4) |
|  |  | PDA, n/N (%) | | | 416/ 3510 (12.0) |
| **Transthoracic echocardiography characteristics** | | | | |  |
|  | LVEF, [Median (IQR)] (n= 3664 ) | | | | 50 (45, 50) |

### IQR: inter quartile range; SD: standard deviation; HDL: high-density lipoprotein; LDL: low-density lipoprotein; CBC diff.: complete blood count with differential; WBC: white blood cell count; RBC: red blood cell count; ALT: Alanine Amino-transferase; AST: Aspartate Trans-aminase; ALP: alkaline phosphatase; BUN: blood urea nitrogen; CRP: C-reactive protein; LMCA: Left main coronary artery; LCX: Left circumflex; LAD: Left anterior descending; RCA: Right coronary artery; PDA: Posterior descending artery; LVEF: Left ventricular ejection fraction
